# Characterization of the glucosylated anthocyanin profile of 27 red grape (*Vitis vinifera* L.) varieties grown in Portugal: insights for climate change adaptation

**DOI:** 10.3389/fpls.2025.1728700

**Published:** 2026-01-26

**Authors:** Miguel Baltazar, Eliana Monteiro, Sandra Pereira, Márcia Carvalho, Elisete Correia, Helena Ferreira, Vânia Silva, Joana Valente, Fernando Alves, Isaura Castro, Berta Gonçalves

**Affiliations:** 1 Centre for the Research and Technology of Agro-Environmental and Biological Sciences (CITAB), University of Trás-os-Montes e Alto Douro (UTAD), Vila Real, Portugal; 2 Institute for Innovation, Capacity Building and Sustainability of Agri-food Production (Inov4Agro), University of Trás-os-Montes e Alto Douro (UTAD), Vila Real, Portugal; 3 Center for Computational and Stochastic Mathematics (CEMAT), Department of Mathematics, University of Trás-os-Montes and Alto Douro, Vila Real, Portugal; 4 Symington Family Estates, Vinhos SA, Vila Nova de Gaia, Portugal

**Keywords:** abiotic stress, berry color, gene expression, intervarietal diversity, varietal selection

## Abstract

**Introduction:**

Climate change poses significant challenges to viticulture, increasing the need for sustainable adaptation strategies such as the identification of resilient *Vitis vinifera* L. varieties.

**Methods:**

This study characterized the anthocyanin content, profile, and color parameters of 27 red grape varieties cultivated under the same terroir in the Douro Demarcated Region over two consecutive years. Berry biochemical analyses, including chromatographic and colorimetric techniques, alongside gene expression of the anthocyanin biosynthesis genes *MybA1, UFGT*, and *OMT*, were conducted to assess varietal and annual variability.

**Results:**

Total anthocyanin content varied significantly among varieties, ranging from 0.14 mg malvidin-3-O-glucoside equivalents per g of dry weight (mg M3G·g^-1^ DW) in ‘Bastardo’ to 8.63 mg M3G·g^-1^ DW in ‘Vinhão’. While most varieties demonstrated increased anthocyanin content in the warmer and drier 2022 season, such as ‘Tinto Cão’ and ‘Touriga Franca’; a few displayed notable declines, notably ‘Vinhão’, highlighting differential responses to abiotic stress. Anthocyanin profiles were dominated by malvidin derivatives, which correlated with enhanced color stability. Nonetheless, cyanidin-3-*O*-glucoside increased in 2022 in some varieties, while delphinidin and petunidin-3-*O*-glucosides decreased. CIELAB parameters indicated darker and higher color saturation in berries in 2022, being associated with increases in total anthocyanin content and malvidin derived compounds. Gene expression analysis of *MybA1, UFGT*, and *OMT* in six varieties revealed different behaviors.

**Discussion:**

Among all varieties under study, stable anthocyanin profiles across years were observed which could suggest increased resilience potential. These findings highlight the interplay between genetic and environmental factors in shaping anthocyanin dynamics, supporting the use of varietal selection as an adaptation strategy to optimize quality, resilience, and sustainability in wine regions under climate change.

## 1 Introduction

Viticulture is a noteworthy socioeconomical activity worldwide, with global wine production in 2023 estimated at 237 million hectoliters, predominantly from European countries (OIV, 2024). Portugal, in particular, ranks as the 10^th^ largest producer and 8^th^ largest exporter of wine worldwide, with the viticultural sector supporting numerous related businesses, including food services and oenotourism (Fraga and Santos, 2017; De Almeida Costa et al., 2021). However, in similarity with other Mediterranean regions, climate change threatens the quantity and quality of grape production in this country, with climate models pointing towards dryer and warmer growth conditions, coupled with a higher frequency of extreme weather events (Dinis et al., 2022; Fonseca et al., 2023).

Over the last decades, the internationalization of the viticultural sector has led to significant losses in grapevine varietal diversity, promoting the use of varieties well accepted by consumers in detriment of lesser-known ones (Díaz-Fernández et al., 2022). This loss of biodiversity is expected to lead to significant decreases in production in most major winegrowing regions under several climate changes scenarios (Morales-Castilla et al., 2020). One such region is the Douro Demarcated Region (DDR) in Portugal, known for having an unique terroir, and acknowledged as the world’s most heterogeneous mountainous wine region (Lourenço-Gomes et al., 2015). Grapevines in the DDR are constantly subjected to stressful conditions. Although this species is known for its resilience to abiotic stress, severe environmental conditions can still impact productivity and berry quality (Bernardo et al., 2018). Given the predicted climate scenarios, several adaptation strategies have been studied and developed in order to mitigate the negative effects of the increased abiotic stress (Santos et al., 2020). One of these approaches, commonly known as varietal selection, focuses on the use of the high intervarietal diversity of grapevine (Baltazar et al., 2025a). This strategy bases itself on the differing behavior of grapevine varieties in order to identify those better adapted or more tolerant to abiotic stress (Morales-Castilla et al., 2020; Santos et al., 2020; Dinis et al., 2022). However, a significant portion of this diversity remains understudied, including in Portugal where over 300 varieties are authorized for wine production (Augusto et al., 2019).

Among the first steps in varietal selection is the characterization of the varieties, such as the assessment of the biochemical profile of the berries (Díaz-Fernández et al., 2022), but also how these respond to changes in environmental conditions (Morales-Castilla et al., 2020; Santos et al., 2020). Red grapes are known to be rich in anthocyanin content, which dictates the color of the wines (Dinis et al., 2024). Moreover, it is known that anthocyanin levels are not only variety-dependent but can also be affected by the climatic conditions of each year (de Rosas et al., 2017, 2022). For instance, higher temperatures and long periods of drought have been shown to disrupt their synthesis and even increase degradation, resulting in less vibrant wines (Movahed et al., 2016; de Rosas et al., 2017; Torres et al., 2017). Despite this, different varieties might respond to environmental stresses in different ways, with some being able to maintain or even enhance their anthocyanin content and grape color (Rocheta et al., 2016; Hewitt et al., 2023). Leveraging this diversity might help viticulturists prioritize varieties with traits associated with higher berry quality under stress conditions, and optimize management practices, promoting the sustainability and economic stability of their productions. Furthermore, understanding mechanisms underlying anthocyanin synthesis in grape berry under abiotic stress conditions can also improve the development of new and more resilient grapevine varieties.

Anthocyanins are mainly present in the epidermal and hypodermal layers of the berry, with teinturier varieties also containing these pigments in the flesh (Ferreira et al., 2019a; Kőrösi et al., 2022). These compounds are known to play a crucial role in the antioxidant defense mechanisms of plants, protecting cellular structures from oxidative damage caused by environmental stressors such as high temperatures and drought (Kaur et al., 2023; Dou et al., 2024). Anthocyanins originate from the flavonoid pathway, and their synthesis begins during veraison. In grape berry, they are mostly present in their 3*-O-*monoglucoside forms, which can be di-substituted and tri-substituted in the lateral B-ring, with the main ones being cyanidin and peonidin (3′-substituted) and delphinidin, petunidin and malvidin (3′, 5′-substituted) (Guidoni et al., 2008; Dai et al., 2011). These originate from the enzymatic activity of flavonoid 3′-hydroxylase (F3′H), leading towards the branch of cyanidin derivates, and flavonoid 3′,5′-hydroxylase (F3′5′H) which leads to the branch of delphinidin derivates (Jeong et al., 2006; Ferreira et al., 2019a). The regulation of this pathway is tightly controlled by transcription factors such as *MybA1*, which leads to the activation of key biosynthetic genes like *UFGT* (UDP-glucose:flavonoid 3*-O-*glucosyltransferase), responsible for stabilizing anthocyanins through glycosylation, and *OMT* (*O-*methyltransferase), which catalyzes the methylation steps critical for anthocyanin diversification and stability (Hugueney et al., 2009; Ali et al., 2011). While anthocyanin composition is known to vary with environmental stressors, the interplay between genetic regulation and abiotic conditions remains underexplored, particularly across diverse grape varieties. As these enzymes compete for the same substrates, a higher activity of one leads to a higher proportion of the respective anthocyanin (Jeong et al., 2006; Falginella et al., 2012). Moreover, as cyanidin, delphinidin, and petunidin contain an *O-*diphenol structure on the B ring, these are more prone to oxidation and therefore less stable, unlike malvidin and peonidin which possess no ortho-positioned hydroxyl groups, with malvidin being even less reactive than peonidin (Wang et al., 2024). These differences in the anthocyanin composition determine the stability and color of wines (Sáenz-Navajas et al., 2011; He et al., 2012), despite some presumed co-pigmentation with other compounds in young wines (Rustioni et al., 2012). Given that different red grape varieties possess different ratios of anthocyanins, characterizing their anthocyanin profile can identify those that produce more stable wines, especially under future climate change scenarios. This kind of knowledge is therefore fundamental for understanding the agronomic and oenological properties of each variety (Baltazar et al., 2025b, 2025c). Prioritizing research on berry quality parameters, like grape color and anthocyanin content, can help viticulturists and oenologists in climate-vulnerable regions to maintain the aesthetic and nutritional qualities of wine and strengthen the overall adaptability and sustainability of the viticulture industry.

As anthocyanins play a critical role in grape and wine quality, several studies have investigated the anthocyanin content of different grapevine varieties under specific terroir conditions. However, further comparative work may still be needed to understand varietal responses under changing climatic scenarios.” Furthermore, it is also important to assess how the expression of genes of the anthocyanin biosynthetic pathway differs among varieties. Thus, the focus of this work was to determine the anthocyanin content, composition and color parameters of 27 red grape varieties, authorized for wine production in Portugal, over two consecutive years (2021 and 2022), while also evaluating the expression of *MybA1, UFGT*, and *OMT* in varieties of cultural relevance and with contrasting physiological behaviors.

## 2 Materials and methods

### 2.1 Plant material, growth conditions, and sampling

This research was carried out in a grapevine variety library located at Quinta do Ataíde, Symington Family Estates (41° 14’ N, 7° 6’ W, 125m), North of Portugal, Vale da Vilariça, Bragança, in the Upper Douro sub-region of the DDR. Plants were established in 2014, with a spacing of 2.2m × 1.0m in a Royat single cordon training system. For this work, 27 red grapevine varieties were sampled, namely ‘Alicante Bouschet’, ‘Alvarelhão’, ‘Aragonez’, ‘Baga’, ‘Bastardo’, ‘Cabernet Sauvignon’, ‘Casculho’, ‘Castelão’, ‘Cornifesto’, ‘Donzelinho Tinto’, ‘Malvasia Preta’, ‘Marufo’, ‘Mourisco de Semente’, ‘Rufete’, ‘Syrah’, ‘Tinta Caiada’, ‘Tinta Carvalha’, ‘Tinta da Barca’, ‘Tinta Francisca’, ‘Tinta Barroca’, ‘Tinto Cão’, ‘Touriga Fêmea’, ‘Touriga Franca’, ‘Touriga Nacional’, ‘Trincadeira’, ‘Vinhão’, and ‘Zinfandel’. Sampling occurred during the 2021 and 2022 growing seasons at the ‘harvest’ stage, following the technological maturity criteria established by the producer (see **Supplementary Tables S1**, **S2** for collection dates, °Brix, and titratable acidity data). Berries were collected by randomly selecting fresh berries from different bunches of three different grapevines of each variety (3 grapevines x 27 varieties). Berries were flash frozen in dry ice, ground to a fine powder under laboratory conditions, and kept at -80°C until analysis.

**Figure 1:**
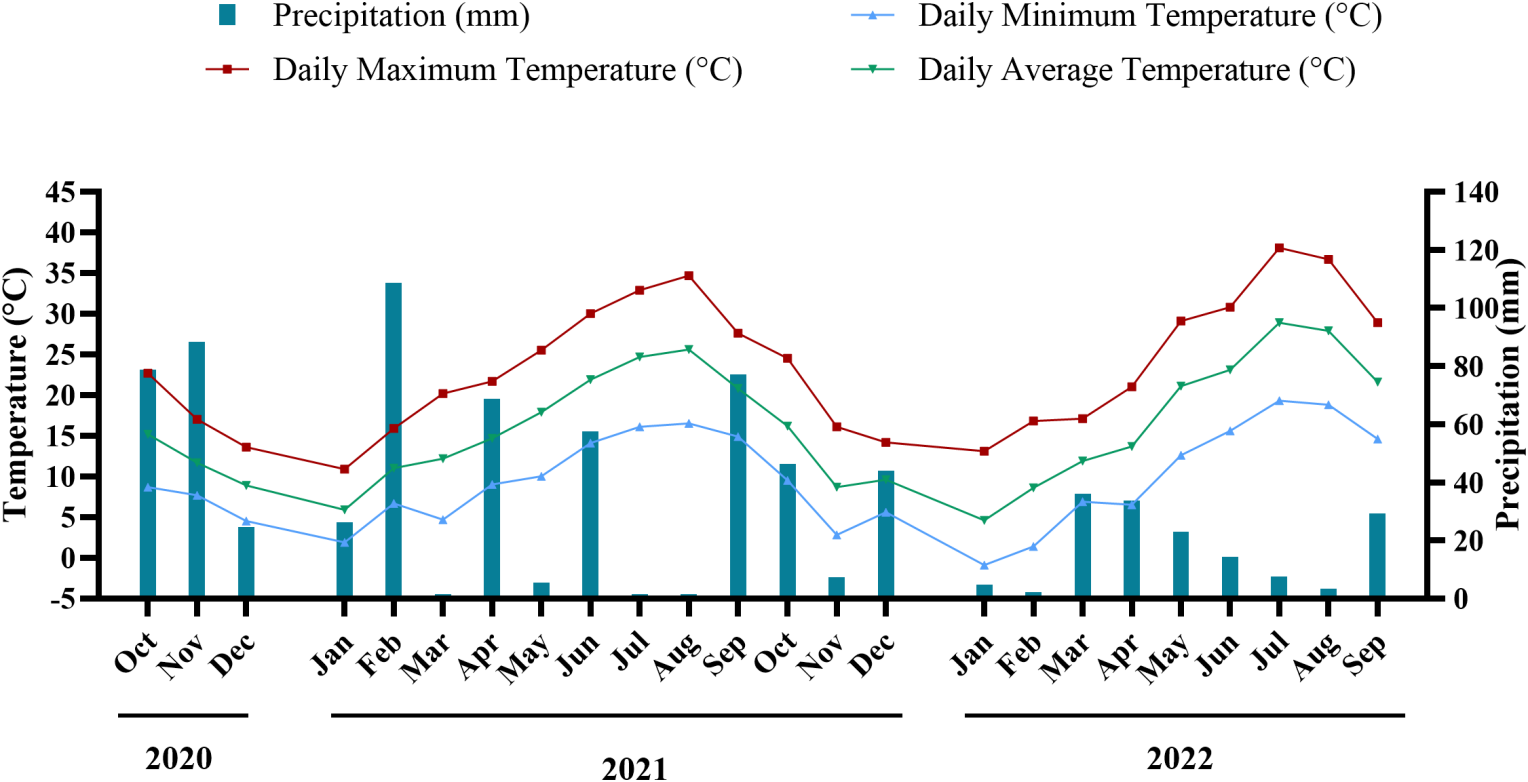
Monthly average values of daily temperature and precipitation in the vineyard from October 2020 to September 2022.

The precipitation and temperature mean values from October 2020 to September 2022 are presented in **Figure 1**. Overall, 2022 was both warmer and drier than 2021. During the first growing season (October 2020–September 2021), the average daily temperature was approximately 15.9°C, starting cooler in the autumn and winter before reaching a daily high of 34.7°C in August. This period was characterized by heavy rainfall, with totals nearing 536mm, primarily falling during the wetter winter months. In contrast, the second growing season (October 2021–September 2022) recorded a slightly higher mean temperature of about 16.3°C, and a higher daily maximum of 38.1°C in July. Additionally, this season experienced markedly less precipitation, with only around 252mm in total, intensifying water stress during key growing periods.

### 2.2 Chemicals and instruments

All chemicals used were of analytical grade (> 99%), water purity was Milli-Q (Merk-Millipore, Darmstadt, Germany), and all solvents were of HPLC grade quality (Fisher Scientific, Loughborough, United Kingdom). The following commercial standards from Extrasynthese (Genay, France) were used for the identification and quantification of anthocyanins: petunidin-3*-O-*glucoside, peonidin 3*-O-*glucoside, delphinidin-3*-O-*glucoside, and malvidin-3*-O-*glucoside.

### 2.3 Color measurements

Berry color was measured using a colorimeter (CR-300, Minolta, Osaka, Japan) following the CIE (Commission International de l’Eclairage, Vienna, Austria) system of 1976 and using the psychometric space (CIELAB). Data was collected on 10 berries of 3 plants of each variety, analyzing 2 sides on each berry (10 berries × 3 plants × 2 measurements). This analysis correlates color values with visual perception, with parameters, being expressed as Cartesian coordinates of lightness (L*), and red-green axis (a*) and yellow-blue axis (b*). L* ranges from 0 (opaque or black) to 100 (transparent or white), a* from red to green (positive values indicate red and negative indicate green), b* from yellow to blue (positive values indicate yellow and negative indicate blue). These values were also transformed into hue (*h°*), expressing color nuance varying between 0°/360° (red), 90° (yellow), 180° (green) and 270° (blue), and chroma (C*), chromaticness relative to the white, where C* = (a*^2^ + b*^2^)^1/2^ (Mclellan et al., 1995; Rolle and Guidoni, 2007; Han et al., 2008). For *h°*, values were determined according to Mclellan et al. (1995), where for positive a* and b* values *h°*= arctan (b*/a*); when either a* or b* are negative, *h°* = 180 + arctan (b*/a*); and with both negative a* and b* h° = 360 + arctan (b*/a*).

### 2.4 Total anthocyanin determination

Total anthocyanin content was determined using the pH differential method established by Lee et al. (2005), with slight modifications. For each grape variety, three independent extracts were obtained and measurements done in triplicate (3 extracts per variety × 3 replicates per extract). These were obtained by adding 5 mL of methanol:hydrochloric acid (99:1, v/v) to 50 mg of lyophilized berries fine powder. These samples were put in an ultrasonic bath for 20 min, centrifuged at 4000 rpm for 15 min at 4°C, and the supernatant collected. For the quantification, 50 μL of extract and 250 μL of either 0.025 mol·L^−1^ potassium chloride (KCl, pH 1.0) or 0.4 mol·L^−1^ sodium acetate buffer (C_2_H_3_NaO_2_, pH 4.5) was added into different microplate wells. Absorbances were read at 520 and 700 nm. Total monomeric anthocyanin content was expressed as milligrams of malvidin-3*-O-*glucoside equivalent (mg M3G) per gram of DW, as per the formula:


Total anthocyanin content=A×MW×df/ϵ×C,

where MW is the molecular weight of malvidin-3*-O-*glucoside (493.43 g.mol^−1^); df the dilution factor; ϵ the molar extinction coefficient of malvidin-3*-O-*glucoside (28,000 L·mol^-1^·cm^-1^); C the concentration of the extracted volume; and A is calculated as:


$$\text{A}={\left({\text{A}}_{520}–{\text{A}}_{700}\right)}_{\text{pH }1.0}–{\left({\text{A}}_{520}–{\text{A}}_{700}\right)}_{\text{pH }4.5}.$$

### 2.5 Anthocyanin profile via HPLC-DAD analysis

For the HPLC-DAD analysis, three independent extracts were obtained for each grape variety, and measurements done in triplicate (3 extracts per variety × 3 replicates per extract). Extraction occurred by weighing 200 mg of fine berry powder and mixing with 1 mL of methanol:hydrochloric acid (99:1, v/v), and setting in an ultrasonic bath for 30 min. Following this, samples were centrifuged at 8000 rpm for 15 min at 4°C, and the supernatant collected and filtered to a vial using a 0.22 µm membrane. These extracts were stored at -20°C until the analysis. HPLC-DAD analyses were carried out using a Thermo Scientific Dionex Ultimate 3000 UHPLC system (Thermo Fisher Scientific, Bremen, Germany) equipped with a quaternary gradient pump, an autosampler, a column oven, and a diode array detector, and based on a reported method (Fraige et al., 2014). Autosampler sample tray was kept at 25°C, and the separation was performed in a ProntoSIL 120-5-C18 ace-EPS column (250 mm × 4.6 mm, 5 μm; BISCHOFF Chromatography, Leonberg, Germany) at 35°C. The mobile phases consisted of water/formic acid/trifluoracetic acid (89.9:10:0.1, v/v, phase A) and acetonitrile formic acid/trifluoracetic acid (89.9:10:0.1,v/v, phase B), with a gradient program as follows (t min:% B): 0 min, 10% B; 5 min:20% B; 25 min:35% B; 30 min:50% B; 35 min:100% B; 45 min:10% B; at a flow rate of 0.8 mL.min^−1^, for a total run time of 45 min. The column was equilibrated for 5 minutes between injections. Injection volume was 10 μL and each extract was analyzed in triplicate. Anthocyanin identification and quantification were made with DAD-chromatograms at 520 nm according to peak retention time and UV-Vis spectra data, by comparison with authentic standards (Extrasynthese, Genay Cedex, France).

### 2.6 Total RNA extraction, cDNA synthesis, and RT-qPCR

Five cultivars ‘Cabernet Sauvignon’, ‘Marufo’, ‘Touriga Nacional’, ‘Touriga Franca’, and ‘Vinhão’ and of *Vitis vinifera* subsp. *vinifera* were selected for gene expressions studies (RT-qPCR). For each variety, total RNA was extracted from three independent biological replicates, with each sample quantified in triplicate (3 biological replicates × 3 technical replicates). Total RNA was isolated from 100 mg of berry tissue using the NZY Plant/Fungi RNA Isolation Kit (Nzytech, Lisbon, Portugal), according to the manufacturer’s instructions. RNA integrity and concentration were estimated through A_260_/A_280_ ratio using a Powerwave XS2 spectrophotometer (BioTek Instruments, Inc., Winooski, USA).

For each sample, total RNA (500 ng) reverse transcription was prepared using a SensiFAST cDNA Synthesis kit (Meridian Bioscience, Memphis, Tennessee, USA), following the manufacturer’s procedure. The differential gene expression of genes involved in the secondary metabolism, namely *OMT*, *UFGT*, and *MybA1* (**Supplementary Table S3**), was carried out via real-time RT-PCR using a StepOnePlus Real Time PCR (Applied Biosystems, Foster City, USA). Each reaction (10 µL) was performed in triplicate, and contained 500 nM of each primer, 2 µL of cDNA (1:5 dilution), 5 µL of PowerUp™ SYBR™ Green Master Mix (Thermo Fisher, Waltham, MA USA), and water up to 10 µL. Thermal cycling conditions consisted of a hold stage at 50°C for 2 min and 95°C for 10 min, followed by 40 cycles: 95°C for 20 s, annealing (variable temperatures, see **Supplementary Table S3**) for 45 s, and 72°C for 30 s. A melting cycle with temperature ranging from 60 to 95°C was used to detect non-specific amplification in cDNA samples. Each of the three biological replicates was used in three technical replicates. Gene transcripts were quantified upon normalization to two reference genes: elongation factor 1-alfa (*EF1α*) (Ferreira et al., 2019a, 2019b) and glyceraldehyde-3-phosphate dehydrogenase (*GAPDH*) (Garrido et al., 2019) by comparing the threshold cycle (Ct) of each target gene with average of the two reference genes Ct. The relative quantification per each gene was calculated according to the 2^−ΔCt^ method, where ΔCt is the difference in threshold cycle between the geometric means of the target and reference genes (*EF1α* and *GAPDH*) (Ferreira et al., 2019a, 2019b).

### 2.7 Statistical analysis

All data sets met the assumptions of variance homogeneity and normality, assessed using Levene’s and Shapiro–Wilk tests, respectively. Data were subjected to two-way ANOVA followed by Tukey’s multiple range test (*p* < 0.05). Statistically significant differences between years and within each variety are indicated as: * - *p* < 0.05; ** - *p* < 0.01; and *** - *p* < 0.001. Statistical analyses were performed using SPSS Statistics for Windows (Version 23.0; IBM Corp., Armonk, NY, USA).

Pearson correlation and principal component analysis (PCA) were performed to explore relationships between variables and reduce data dimensionality. PCA components with eigenvalues greater than 1 were retained, and variable contributions were interpreted based on factor loadings. These analyses were carried out using GraphPad Prism version 10.4.0 (GraphPad Software, San Diego, CA, USA).

## 3 Results

### 3.1 Total anthocyanin content

**Figure 2:**
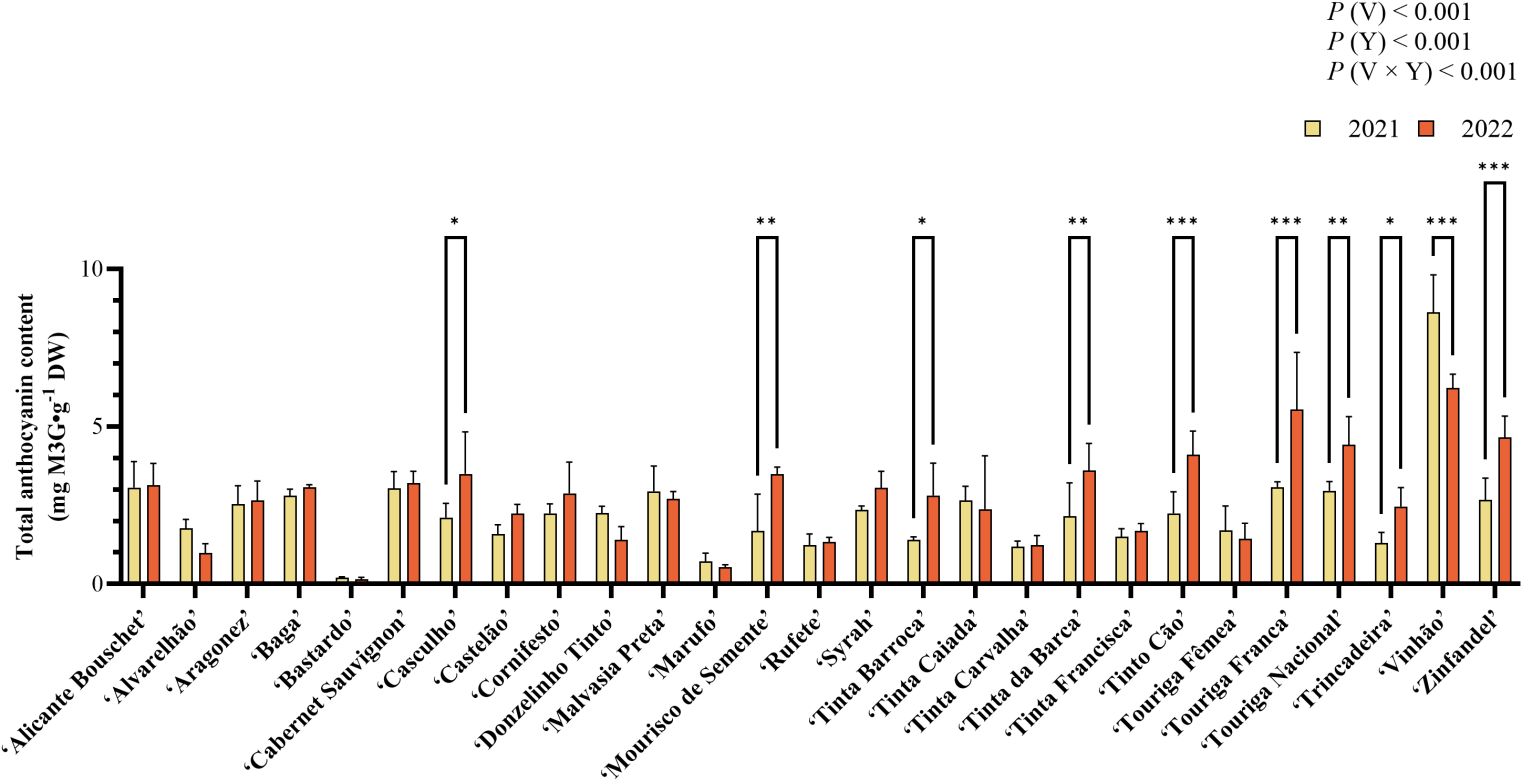
Total anthocyanin content (TAC) of the varieties under study expressed as mg M3G.g^-1^ DW. For statistically significant differences between years within varieties: *p < 0.05; **p < 0.01; ***p < 0.001. V, variety; Y, year.

The results from the quantification of total anthocyanin content are presented in **Figure 2**. Statistically significant differences (*p* < 0.001) were observed for all factors: variety, year, and the interaction variety × year. The highest value for total anthocyanin content was observed in ‘Vinhão’ in both years, with a concentration of 8.63 mg M3G.g^-1^ DW in 2021 and 6.22 mg M3G.g^-1^ DW in 2022. On the other hand, the lowest content of total anthocyanins in both years was observed in ‘Bastardo’ which presented values of 0.21 and 0.14 mg M3G.g^-1^ DW in 2021 and 2022, respectively. From 2021 to 2022, ‘Casculho’, ‘Mourisco de Semente’, ‘Tinta Barroca’, ‘Tinta da Barca’, ‘Tinto Cão’, ‘Touriga Franca’, ‘Touriga Nacional’, ‘Trincadeira’, and ‘Zinfandel’ presented statistically significant increases (*p* < 0.05) in anthocyanin content, while ‘Vinhão’ was the only one with a significant decrease (*p* < 0.001) in concentration.

### 3.2 Anthocyanin profile

Six anthocyanins were identified and quantified in the 27 grape varieties in analysis for both years, with the results being expressed as percentage of total anthocyanins in **Table 1**. The identified anthocyanins were delphinidin-3*-O-*glucoside, cyanidin-3*-O-*glucoside, petunidin-3*-O-*glucoside, peonidin-3*-O-*glucoside, malvidin-3*-O-*glucoside, and malvidin-3*-O-*(6*-O-*trans-p-coumaroyl-glucoside). Among these, the most predominant anthocyanin was malvidin-3*-O-*glucoside, and the less predominant one was cyanidin-3*-O-*glucoside. The highest percentage of delphinidin-3*-O-*glucoside was observed for ‘Vinhão’ in 2021 (24.98 ± 1.034%), and the lowest in ‘Tinta Barroca’ in 2022 (1.79 ± 0.280%). Despite cyanidin-3*-O-*glucoside being found in low percentages in most varieties, variety ‘Bastardo’ presented high percentages in both years (21.13 ± 2.034% in 2021 and 22.86 ± 3.635% in 2022), contrasting with the lowest levels detected in ‘Touriga Nacional’ (0.08 ± 0.011% in 2021).

**Table 1:** Concentration of the anthocyanins found in the 27 grape varieties under study.

Variety	Year	Delphinidin-3*-O-*glucoside	Cyanidin-3*-O-*glucoside	Petunidin-3*-O-*glucoside	Peonidin-3*-O-*glucoside	Malvidin-3*-O-*glucoside	Malvidin-3*-O-*(6*-O-*trans-p-coumaroyl-glucoside)
‘Alicante Bouschet’	2021	4.57 ± 1.175*	1.99 ± 1.093	7.06 ± 2.070*	24.46 ± 3.754***	50.09 ± 1.054*	11.83 ± 5.281**
	2022	2.09 ± 0.372*	0.34 ± 0.052	4.16 ± 0.178*	11.66 ± 3.170***	61.67 ± 3.401*	20.14 ± 0.440**
‘Alvarelhão’	2021	9.23 ± 0.439	13.7 ± 1.147*	7.70 ± 0.651	43.32 ± 1.958***	18.84 ± 2.311*	7.20 ± 0.616***
	2022	9.51 ± 0.547	7.22 ± 1.803*	7.95 ± 0.168	27.34 ± 4.918***	29.55 ± 6.891*	21.19 ± 1.917***
‘Aragonez’	2021	24.62 ± 1.432***	1.81 ± 0.308	17.99 ± 1.245***	3.10 ± 0.669	36.46 ± 1.147	16.03 ± 2.268***
	2022	17.55 ± 2.584***	1.12 ± 0.546	13.85 ± 1.001***	3.00 ± 0.591	37.12 ± 1.982	27.37 ± 2.969***
‘Baga’	2021	9.43 ± 2.861*	1.10 ± 0.601	10.36 ± 1.524	9.07 ± 1.178	69.56 ± 4.189	0.49 ± 0.291
	2022	6.95 ± 2.145*	1.95 ± 1.595	8.07 ± 1.330	8.02 ± 1.459	70.68 ± 11.860	0.29 ± 0.105
‘Bastardo’	2021	17.55 ± 1.300	21.13 ± 2.034*	16.55 ± 2.453	10.68 ± 3.206*	25.24 ± 5.669	12.18 ± 1.308***
	2022	18.32 ± 3.351	22.86 ± 3.635*	18.16 ± 2.374	14.36 ± 3.525*	28.29 ± 10.600	22.86 ± 1.626***
‘Cabernet Sauvignon’	2021	13.35 ± 0.768***	2.82 ± 0.578***	13.14 ± 1.167***	4.48 ± 0.391	51.03 ± 6.692	12.18 ± 1.502***
	2022	5.38 ± 1.245***	0.18 ± 0.130***	5.58 ± 0.921***	5.12 ± 0.651	57.11 ± 0.894	26.63 ± 1.643***
‘Casculho’	2021	4.12 ± 0.970	0.55 ± 0.179	5.46 ± 0.437	5.11 ± 0.948	58.38 ± 3.208	24.87 ± 5.158
	2022	4.09 ± 0.988	0.51 ± 0.529	5.53 ± 0.951	3.50 ± 1.047	58.40 ± 2.436	27.99 ± 5.874
‘Castelão’	2021	13.16 ± 3.525**	2.03 ± 1.184	12.66 ± 2.044**	11.15 ± 3.370	51.42 ± 8.180	9.58 ± 1.609**
	2022	9.10 ± 0.444**	1.90 ± 0.538	9.29 ± 0.556**	7.61 ± 3.038	53.29 ± 2.098	18.81 ± 1.661**
‘Cornifesto’	2021	7.00 ± 1.053	0.85 ± 0.017	8.51 ± 0.789	6.76 ± 1.007	76.59 ± 3.597	0.83 ± 0.170
	2022	7.66 ± 0.442	1.13 ± 0.308	9.57 ± 0.164	6.29 ± 0.538	75.06 ± 0.437	0.39 ± 0.093
‘Donzelinho Tinto’	2021	18.47 ± 2.188	16.40 ± 3.959***	14.65 ± 1.905	27.43 ± 5.147***	22.72 ± 3.840**	1.47 ± 0.015
	2022	18.24 ± 1.442	7.67 ± 0.062***	15.37 ± 0.243	14.99 ± 3.154***	38.93 ± 3.761**	5.90 ± 1.740
‘Malvasia Preta’	2021	8.48 ± 1.487**	0.72 ± 0.149	10.00 ± 1.478**	3.33 ± 0.723	49.72 ± 1.717	27.93 ± 4.754***
	2022	4.84 ± 1.217**	0.42 ± 0.260	6.28 ± 1*.393*	1.87 ± 1.084	42.66 ± 1.763	44.35 ± 3.363***
‘Marufo’	2021	7.31 ± 0.680	0.44 ± 0.176**	10.6 ± 2.888	16.91 ± 4.807***	45.05 ± 9.902	29.49 ± 5.165***
	2022	7.20 ± 1.457	3.02 ± 1.317**	8.91 ± 2.875	4.78 ± 2.343***	45.23 ± 2.623	37.37 ± 3.795***
‘Mourisco de Semente’	2021	6.79 ± 2.391	3.80 ± 1.850**	8.61 ± 1.120	7.05 ± 2.856	64.29 ± 15.031***	18.85 ± 1.630***
	2022	5.77 ± 1.424	1.61 ± 0.649**	6.69 ± 0.779	10.54 ± 1.587	44.64 ± 9.209***	32.88 ± 4.270***
‘Rufete’	2021	6.84 ± 1.516	2.21 ± 0.980	7.98 ± 2.639	2.50 ± 1.062	60.72 ± 13.154***	30.77 ± 2.983
	2022	8.35 ± 2.589	1.09 ± 0.903	8.74 ± 1.965	3.43 ± 0.758	43.32 ± 1.005***	35.66 ± 5.350
‘Syrah’	2021	4.82 ± 0.157	0.69 ± 0.486	7.24 ± 0.308	2.94 ± 0.717	55.42 ± 1.322	28.88 ± 1.773
	2022	5.61 ± 1.301	0.82 ± 0.690	7.28 ± 2.256	3.29 ± 1.038	51.67 ± 3.140	31.33 ± 6.118
‘Tinta Barroca’	2021	2.20 ± 0.429	2.52 ± 2.084**	1.60 ± 0.084	4.04 ± 2.727	58.18 ± 10.383***	34.64 ± 2.796***
	2022	1.79 ± 0.280	0.25 ± 0.066**	2.55 ± 0.435	2.54 ± 0.303	33.77 ± 1.546***	59.11 ± 2.316***
‘Tinta Caiada’	2021	6.51 ± 0.849***	2.26 ± 1.090	16.66 ± 1.828	1.86 ± 0.450	46.66 ± 10.570	24.14 ± 2.266
	2022	15.05 ± 1.743***	2.60 ± 0.807	18.12 ± 0.925	2.63 ± 1.040	39.36 ± 1.941	22.24 ± 4.365
‘Tinta Carvalha’	2021	7.49 ± 1.213*	2.01 ± 0.836	8.49 ± 1.147*	18.85 ± 4.635***	58.91 ± 9.080	8.03 ± 3.311***
	2022	10.11 ± 1.699*	3.32 ± 0.654	10.81 ± 1.422*	10.96 ± 1.648***	52.60 ± 7.034	20.02 ± 2.488***
‘Tinta da Barca’	2021	5.34 ± 3.482*	2.69 ± 0.906	8.44 ± 0.839*	8.70 ± 2.552	62.22 ± 10.675*	16.23 ± 1.284***
	2022	3.88 ± 0.342*	1.09 ± 0.648	5.85 ± 0.283*	5.46 ± 1.775	50.53 ± 3.805*	33.19 ± 3.517***
‘Tinta Francisca’	2021	5.54 ± 1.271**	0.29 ± 0.250	6.29 ± 1.065*	4.23 ± 0.646	52.79 ± 2.062	30.87 ± 0.957
	2022	2.23 ± 1.035**	1.61 ± 0.187	3.98 ± 0.580*	3.27 ± 0.880	61.47 ± 7.299	31.1 ± 4.085
‘Tinto Cão’	2021	5.56 ± 0.039	0.67 ± 0.676	6.55 ± 0.191	3.58 ± 1.188	34.04 ± 0.901	47.80 ± 5.270
	2022	3.83 ± 0.323	0.89 ± 0.024	5.97 ± 1.546	2.41 ± 1.033	39.13 ± 4.113	47.02 ± 8.373
‘Touriga Fêmea’	2021	8.19 ± 0.435	0.73 ± 0.426	9.07 ± 0.360	3.06 ± 1.935	60.15 ± 9.603	21.95 ± 3.334***
	2022	7.75 ± 2.691	0.09 ± 0.116	7.94 ± 1.051	2.50 ± 0.855	50.46 ± 8.776	33.63 ± 6.749***
‘Touriga Franca’	2021	7.16 ± 1.481***	1.33 ± 0.115	9.17 ± 2.784***	9.38 ± 3.116***	52.95 ± 6.399*	24.92 ± 2.725***
	2022	2.70 ± 1.280***	0.44 ± 0.191	3.95 ± 1.382***	2.22 ± 1.094***	42.28 ± 6.917*	53.38 ± 6.037***
‘Touriga Nacional’	2021	4.61 ± 0.213	0.08 ± 0.011	5.62 ± 0.353	2.80 ± 0.173	45.01 ± 1.152	41.85 ± 1.951
	2022	3.17 ± 0.883	0.18 ± 0.031	4.14 ± 1.227	2.91 ± 0.878	42.46 ± 2.233	47.05 ± 2.745
‘Trincadeira’	2021	9.50 ± 0.912	1.56 ± 0.798	13.23 ± 0.508	7.98 ± 3.905*	62.42 ± 6.331	10.62 ± 1.638
	2022	10.41 ± 2.199	0.36 ± 0.099	11.53 ± 1.398	3.49 ± 0.166*	60.02 ± 8.056	15.81 ± 3.069
‘Vinhão’	2021	24.98 ± 1.034***	2.52 ± 0.345*	16.78 ± 0.850***	4.52 ± 0.603	45.78 ± 1.832**	5.43 ± 0.451***
	2022	8.99 ± 1.867***	0.66 ± 0.264*	8.93 ± 1.669***	3.61 ± 0.410	62.20 ± 3.800**	15.61 ± 3.354***
‘Zinfandel’	2021	8.13 ± 0.992*	1.87 ± 0.699	11.59 ± 2.271***	3.80 ± 1.019	49.81 ± 3.386	28.60 ± 0.616***
	2022	5.57 ± 0.950*	0.30 ± 0.050	7.54 ± 1.223***	2.76 ± 0.674	46.5 ± 6.276	42.22 ± 3.083***
*P* (V)		< 0.001	< 0.001	< 0.001	< 0.001	< 0.001	< 0.001
*P* (Y)		< 0.001	< 0.001	< 0.001	< 0.001	n.s.	< 0.001
*P* (V × Y)		< 0.001	< 0.001	< 0.001	< 0.001	< 0.001	< 0.001

Results are expressed as mean values ± standard deviation of percentage (%). For statistically significant differences between years within varieties: *p < 0.05; **p < 0.01; ***p < 0.001. V, variety; Y, year.

Petunidin-3*-O-*glucoside percentages presented higher variability among varieties, with the highest concentration being found in ‘Bastardo’ in 2022 (18.16 ± 2.374%), and the lowest in ‘Tinta Barroca’ in 2021 (1.60 ± 0.084%). In a similar manner, peonidin-3*-O-*glucoside ranged from 1.86 ± 0.450% in ‘Tinta Caiada’ in 2021 to 43.32 ± 1.958% in ‘Alvarelhão’, also in 2021. Regarding all anthocyanins, malvidin derived compounds, namely malvidin-3*-O-*glucoside and malvidin-3*-O-*(6*-O-*trans-p-coumaroyl-glucoside) were the predominant compounds in most varieties.

Among these two anthocyanins, the highest percentage of malvidin-3*-O-*glucoside was 76.59 ± 3.597% in ‘Cornifesto’ in 2021, with the lowest being 18.84 ± 2.311% in ‘Alvarelhão’ in 2021. On the other hand, malvidin-3*-O-*(6*-O-*trans-p-coumaroyl-glucoside) demonstrated the widest range, with ‘Tinta Barroca’ exhibiting the highest concentration (59.11 ± 2.316%) and ‘Baga’ the lowest (0.29 ± 0.105%) in 2022. The ANOVA analysis indicated significant effects of variety, year, and the interaction between variety × year (*p* < 0.001) in almost all anthocyanins, with the only exception being the non-significance of year effect in the percentage of malvidin-3*-O-*glucoside. In fact, delphinidin-, cyanidin-, petunidin-, and peonidin-3*-O-*glucosides revealed mostly statistically significant decreases from 2021 to 2022, while malvidin-3*-O-*(6*-O-*trans-p-coumaroyl-glucoside) predominantly increased in all varieties. As indicated by the non-significance of the year effect, the percentage of malvidin-3*-O-*glucoside remained largely unchanged between years, with very few exceptions, namely the statistically significant increases (*p* < 0.05) in ‘Alicante Bouschet’, ‘Donzelinho Tinto’, and ‘Vinhão’, and the decreases in ‘Rufete’, ‘Tinta Barroca’, and ‘Tinta da Barca’.

### 3.3 CIELAB parameters

CIELAB parameters L*, a*, and b* are presented in **Table 2**, while Chroma (C*) and hue (*h°*) are presented in **Table 3**. In all these parameters, statistical analysis indicated that most of the variation observed was due to the variety factor (above 50%), except for L*, in which the year factor accounted for 41.05% of the variation observed.

**Table 2:** Chromatic parameters lightness (L*) and coordinates a* and b* of the varieties under study.

Variety	Lightness (L*)		a*		b*	
	2021	2022	2021	2022	2021	2022
‘Alicante Bouschet’	30.02 ± 3.166***	33.36 ± 4.614***	0.03 ± 0.444**	-0.32 ± 0.590**	-3.039 ± 1.38	-3.51 ± 1.630
‘Alvarelhão’	29.31 ± 4.059***	38.37 ± 4.085***	0.17 ± 0.626***	1.88 ± 1.608***	-3.95 ± 1.712	-3.98 ± 1.436
‘Aragonez’	28.35 ± 1.745***	31.16 ± 2.902***	-0.16 ± 0.236	-0.27 ± 0.349	-2.39 ± 0.843**	-3.26 ± 1.179**
‘Baga’	23.53 ± 2.161***	33.37 ± 3.142***	0.64 ± 0.499***	-0.20 ± 0.531***	-1.36 ± 1.001***	-4.29 ± 1.510***
‘Bastardo’	29.54 ± 2.732***	32.88 ± 5.544***	0.48 ± 0.468***	2.06 ± 1.665***	-2.93 ± 1.245	-3.38 ± 1.734
‘Cabernet Sauvignon’	33.07 ± 4.249***	39.39 ± 4.536***	-0.85 ± 0.342	-0.71 ± 0.453	-5.28 ± 1.652	-5.23 ± 1.550
‘Casculho’	28.01 ± 4.446***	33.75 ± 3.900***	-0.35 ± 0.596	-0.62 ± 0.410	-3.49 ± 2.202*	-4.13 ± 1.587*
‘Castelão’	28.85 ± 2.749**	30.74 ± 3.005**	0.21 ± 0.410	0.10 ± 0.669	-2.60 ± 1.208**	-3.32 ± 1.381**
‘Cornifesto’	28.87 ± 2.706**	30.75 ± 3.487**	0.15 ± 0.432	0.16 ± 0.571	-2.41 ± 1.222	-2.91 ± 1.327
‘Donzelinho-Tinto’	34.20 ± 3.576	34.66 ± 3.207	-0.14 ± 0.548***	1.01 ± 0.717***	-4.52 ± 1.616***	-3.55 ± 1.241***
‘Malvasia Preta’	30.02 ± 2.320	30.25 ± 2.562	-0.30 ± 0.276***	0.22 ± 0.608***	-3.17 ± 1.013	-2.67 ± 1.213
‘Marufo’	29.89 ± 2.531***	33.09 ± 2.943***	0.25 ± 0.464***	1.23 ± 0.727***	-2.90 ± 1.058	-2.90 ± 1.268
‘Mourisco-de-Semente’	29.47 ± 2.663*	31.10 ± 3.616*	-0.16 ± 0.325*	0.14 ± 0.501*	-2.77 ± 1.074	-2.54 ± 1.198
‘Rufete’	28.10 ± 2.759***	32.05 ± 3.574***	0.06 ± 0.314	-0.01 ± 0.552	-2.18 ± 1.081***	-3.47 ± 1.418***
‘Syrah’	25.81 ± 3.722***	35.30 ± 3.433***	-0.18 ± 0.598**	-0.54 ± 0.380**	-3.13 ± 2.018***	-4.90 ± 1.296***
‘Tinta Barroca’	32.58 ± 4.027***	39.46 ± 4.538***	-0.89 ± 0.361	-0.85 ± 0.400	-4.92 ± 1.420**	-5.78 ± 1.753**
‘Tinta Caiada’	26.68 ± 3.485***	37.28 ± 4.753***	0.67 ± 1.754	0.45 ± 1.080	-2.55 ± 1.886***	-4.39 ± 1.643***
‘Tinta Carvalha’	26.01 ± 3.582***	33.19 ± 3.713***	0.17 ± 0.641	0.00 ± 0.437	-2.96 ± 1.694	-3.43 ± 1.239
‘Tinta da Barca’	24.22 ± 3.268***	29.31 ± 2.908***	0.09 ± 0.575	-0.12 ± 0.339	-1.84 ± 1.492***	-2.78 ± 1.224***
‘Tinta Francisca’	28.56 ± 2.741***	33.32 ± 3.721***	-0.14 ± 0.321	-0.22 ± 0.475	-2.59 ± 1.078***	-3.73 ± 1.317***
‘Tinto Cão’	31.95 ± 3.344	31.60 ± 3.327	-0.28 ± 0.333*	-0.02 ± 0.370*	-2.95 ± 1.255	-2.73 ± 1.197
‘Touriga Fêmea’	33.22 ± 4.323***	37.32 ± 3.790***	0.61 ± 2.344	0.50 ± 1.487	-4.88 ± 1.704	-4.57 ± 1.452
‘Touriga Franca’	30.35 ± 3.668*	31.67 ± 3.838 *	-0.13 ± 0.459	-0.17 ± 0.625	-2.60 ± 1.425	-2.76 ± 1.308
‘Touriga Nacional’	28.53 ± 2.794***	32.25 ± 3.572 ***	-0.04 ± 0.322	-0.29 ± 0.382	-2.44 ± 1.422	-3.14 ± 1.300**
‘Trincadeira’	27.39 ± 2.000***	34.74 ± 4.688***	0.09 ± 0.302***	-0.37 ± 0.420***	-1.99 ± 0.791***	-3.99 ± 1.381***
‘Vinhão’	28.65 ± 4.519***	38.35 ± 2.800***	-0.47 ± 0.472	-0.57 ± 0.339	-3.53 ± 1.884***	-5.66 ± 1.076***
‘Zinfandel’	29.61 ± 3.209***	34.83 ± 3.742***	-0.82 ± 0.387	-0.69 ± 0.330	-5.09 ± 1.576	-4.62 ± 1.313
*P* (V)	< 0.001		< 0.001		< 0.001	
*P* (Y)	< 0.001		< 0.001		< 0.001	
*P* (V × Y)	< 0.001		< 0.001		< 0.001	

Results are expressed as mean values ± standard deviation. For statistically significant differences between years within varieties: *p < 0.05; **p < 0.01; ***p < 0.001. V, variety; Y, year.

**Table 3:** Chromatic parameters chroma (C*) and hue angle (*h°*) of the varieties under study.

Variety	Chroma (C*)		Hue (*h*°)	
	2021	2022	2021	2022
‘Alicante Bouschet’	3.08 ± 0.506	3.57 ± 1.078	252.35 ± 38.615	243.54 ± 46.92
‘Alvarelhão’	4.04 ± 0.253	4.74 ± 0.740	219.85 ± 10.204	222.78 ± 25.567
‘Aragonez’	2.41 ± 0.18	3.28 ± 0.333	213.95 ± 18.472*	270.04 ± 35.466*
‘Baga’	1.73 ± 0.325***	4.34 ± 0.055***	243.54 ± 23.331	237.56 ± 18.468
‘Bastardo’	3.05 ± 0.393**	4.44 ± 0.130**	287.84 ± 26.638***	202.12 ± 18.422***
‘Cabernet Sauvignon’	5.35 ± 0.808	5.31 ± 0.098	187.16 ± 5.103	193.27 ± 13.521
‘Casculho’	3.67 ± 1.132	4.18 ± 0.652	222.41 ± 27.111	237.62 ± 18.405
‘Castelão’	2.67 ± 0.287	3.43 ± 0.331	335.08 ± 13.528	344.07 ± 13.512
‘Cornifesto’	2.47 ± 0.579	3.00 ± 0.443	240.53 ± 27.082***	344.02 ± 5.144***
‘Donzelinho-Tinto’	4.57 ± 1.035	3.81 ± 0.317	308.45 ± 5.052	344.05 ± 25.735
‘Malvasia Preta’	3.19 ± 0.147	2.78 ± 0.379	274.27 ± 16.42	243.45 ± 62.056
‘Marufo’	2.97 ± 0.441	3.35 ± 0.382	237.6 ± 97.882	278.96 ± 46.123
‘Mourisco-de-Semente’	2.79 ± 0.236	2.62 ± 0.727	208.25 ± 0.862	207.98 ± 17.731
‘Rufete’	2.21 ± 0.457**	3.54 ± 0.640**	290.77 ± 56.321	280.21 ± 21.308
‘Syrah’	3.33 ± 0.497**	4.94 ± 0.056**	208.02 ± 15.356**	281.97 ± 60.432**
‘Tinta Barroca’	2.62 ± 0.422*	3.76 ± 0.204*	234.63 ± 26.575*	287.83 ± 30.737*
‘Tinta Caiada’	5.01 ± 0.455	5.85 ± 0.454	299.57 ± 22.268*	246.49 ± 25.572*
‘Tinta Carvalha’	3.19 ± 0.693**	4.65 ± 0.438**	184.35 ± 5.118	190.28 ± 8.878
‘Tinta da Barca’	3.11 ± 0.594	3.47 ± 0.489	269.97 ± 17.909	273.02 ± 18.463
‘Tinta Francisca’	2.13 ± 0.110	2.81 ± 0.982	281.92 ± 28.604	267.13 ± 13.523
‘Tinto Cão’	2.98 ± 0.589	2.76 ± 0.262	305.53 ± 8.842*	258.22 ± 20.418*
‘Touriga Fêmea’	5.41 ± 0.621	4.80 ± 0.796	287.82 ± 31.878***	344.15 ± 25.678***
‘Touriga Franca’	2.65 ± 0.742	2.83 ± 0.473	314.06 ± 8.996*	231.7 ± 22.315*
‘Touriga Nacional’	2.46 ± 0.556	3.17 ± 0.612	269.9 ± 15.35**	193.25 ± 20.48**
‘Trincadeira’	2.03 ± 0.403***	4.03 ± 0.562***	184.36 ± 5.121	190.3 ± 8.86
‘Vinhão’	3.67 ± 0.126***	5.69 ± 0.163***	207.75 ± 17.574	193.26 ± 13.523
‘Zinfandel’	5.18 ± 0.575	4.68 ± 0.025	187.33 ± 5.112	184.38 ± 5.121
*P* (V)	< 0.001		< 0.001	
*P* (Y)	< 0.001		n.s.	
*P* (V × Y)	< 0.001		< 0.001	

Results are expressed as mean values ± standard deviation. For statistically significant differences between years within varieties: *p < 0.05; **p < 0.01; ***p < 0.001. V, variety; Y, year.

For L*, ANOVA results revealed statistically significant effects (*p* < 0.001) for variety, year, and the interaction between variety × year. In 2021, L* values ranged from 23.53 to 34.20 from ‘Baga’ and ‘Donzelinho-Tinto’ respectively, while in 2022 values ranged from 29.31 in ‘Tinta Francisca’ to 39.46 in ‘Tinta Caiada’. Statistically significant increases (*p* < 0.05) were observed in almost all varieties, with the exception of ‘Donzelinho-Tinto’, ‘Malvasia Preta’, and ‘Tinto Cão’, with this last one presenting the only decrease in L*. As previously mentioned, the year factor accounted for the largest portion of variation (41.05%), followed by the variety (31.01%), confirming the observed trend of increased lightness in 2022 across most varieties. When it comes to the red-green balance parameter, a*, positive values indicate shifts towards red and negative values towards green. ANOVA revealed statistically significant effects (*p* < 0.01) for variety and the interaction variety × year, but not for year. Statistically significant increases (*p* < 0.05) in a* values were observed for varieties ‘Alvarelhão’, ‘Bastardo’, ‘Donzelinho-Tinto’, ‘Malvasia Preta’, ‘Marufo’, ‘Mourisco-de-Semente’, and ‘Tinto Cão’, while varieties ‘Alicante Bouschet’, ‘Aragonez’, ‘Baga’, ‘Casculho’, ‘Syrah’, ‘Tinta Francisca’, ‘Touriga Nacional’, ‘Trincadeira’, and ‘Zinfandel’ revealed a statistically significant decrease (*p* < 0.05) in values, indicating a shift towards green (**Table 2**). Regarding the yellow-blue balance, b*, statistically significant effects (*p* < 0.01) were observed for all factors. All varieties presented a negative b* value (**Table 2**), indicating a shift towards blueish tones, with, ‘Aragonez’, ‘Baga’, ‘Castelão’, ‘Cornifesto’, ‘Rufete’, ‘Syrah’, ‘Tinta Caiada’, ‘Tinta Carvalha’, ‘Tinta Francisca’, ‘Tinta Barroca’, ‘Touriga Nacional’, ‘Trincadeira’, and ‘Vinhão’ showing a statistically significant decrease (*p* < 0.05). Only ‘Donzelinho-Tinto’ and ‘Malvasia Preta’ presented significant (*p* < 0.05) increases in b* value, however these were still negative.

Chroma (C*) and hue (*h°*) values are presented in **Table 3**. For both of these CIELAB parameters, statistically significant effects (*p* < 0.001) were observed for variety and the interaction between variety × year, while the year was only significant for C* values. In most varieties there was an increase in C* between both years in study, indicating grapes had a more saturated or intense color in 2022 than in 2021. Nonetheless, only the varieties ‘Baga’, ‘Bastardo’, ‘Rufete’, ‘Syrah’, Tinta Barroca, ‘Tinta Carvalha’, ‘Trincadeira’, and ‘Vinhão’ presented a statistically significant increase (*p* < 0.05) from 2021 to 2022. Comparatively to C*, changes in *h°* were a lot more heterogeneous, with no evident pattern. In 2021, values ranged from 184.35° in ‘Tinta Carvalha’ to 335.08° in ‘Castelão’ respectively, while in 2022 values ranged from 184.38° in ‘Zinfandel’ to 344.15° in ‘Touriga Fêmea’. Statistically significant increases (*p* < 0.05) from 2021 to 2022 were observed in ‘Touriga Nacional’, ‘Syrah’, ‘Tinta Barroca’, and ‘Tinta Caiada’, suggesting a shift toward warmer tones, indicating the berries presented warmer tones; while *h*° values decreased in ‘Cornifesto’, ‘Tinto Cão’, ‘Touriga Franca’, and ‘Touriga Fêmea’ (*p* < 0.05) (**Table 3**) indicating a shift towards cooler tones.

### 3.4 Gene expression

**Figure 3:**
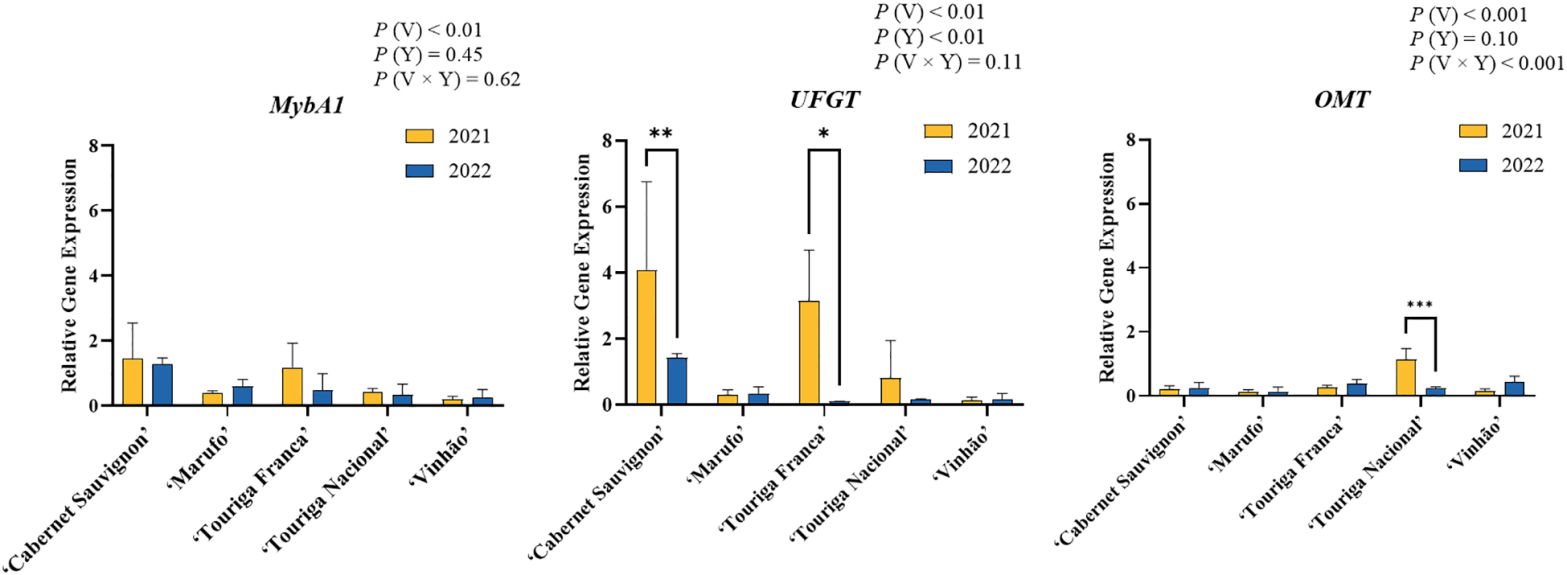
Relative gene expression of *MybA1*, *UFGT*, and *OMT* in berries between the years of 2021 and 2022. Results are expressed as mean values ± standard deviation. For statistically significant differences between years within varieties: *p < .0.05; **p < 0.01; ***p < 0.001. V, variety; Y, year.

In regard to gene expression, three genes involved in the anthocyanin biosynthetic pathway were studied, namely *UFGT* and *OMT*, which encode for UDP-glucose:flavonoid 3*-O-*glucosyltransferase and O-methyltransferase respectively, and *MybA1* which encodes for a transcription factor. Out of all varieties under study, ‘Cabernet Sauvignon’, ‘Marufo’, ‘Touriga Franca’, ‘Touriga Nacional’, and ‘Vinhão’ were selected based on their national and international relevance and/or on their contrasting behavior. The variation in expression for each gene is presented in **Figure 3**. For *MybA1*, ANOVA revealed the variety factor to be the only significant source of variation (48.05%, *p* < 0.01), while neither the interaction nor the year factor revealed significant results. Moreover, no statistically significant differences were observed for each variety between both years, with gene expression levels remaining stable between 2021 and 2022.

In the case of *UFGT*, significant differences were observed for both the variety and the year factors (*p* < 0.01), with variety contributing the most to the variation observed. Among the five varieties selected, statistically significant decreases were observed between years for ‘Cabernet Sauvignon’ and ‘Touriga Franca’ (*p* < 0.05), while ‘Marufo’, ‘Touriga Nacional’, and ‘Vinhão’ remained stable.

Finally, *OMT* gene expression results revealed that the interaction factor variety × year accounted for the majority of the variation (50.95%), followed by variety (44.08%), with both being statistically significant (*p* < 0.001). Differences between years were only statistically significant observed for ‘Touriga Nacional’, which decreased in *OMT* gene expression in 2022 (*p* < 0.001).

### 3.5 Pearson correlation

**Figure 4:**
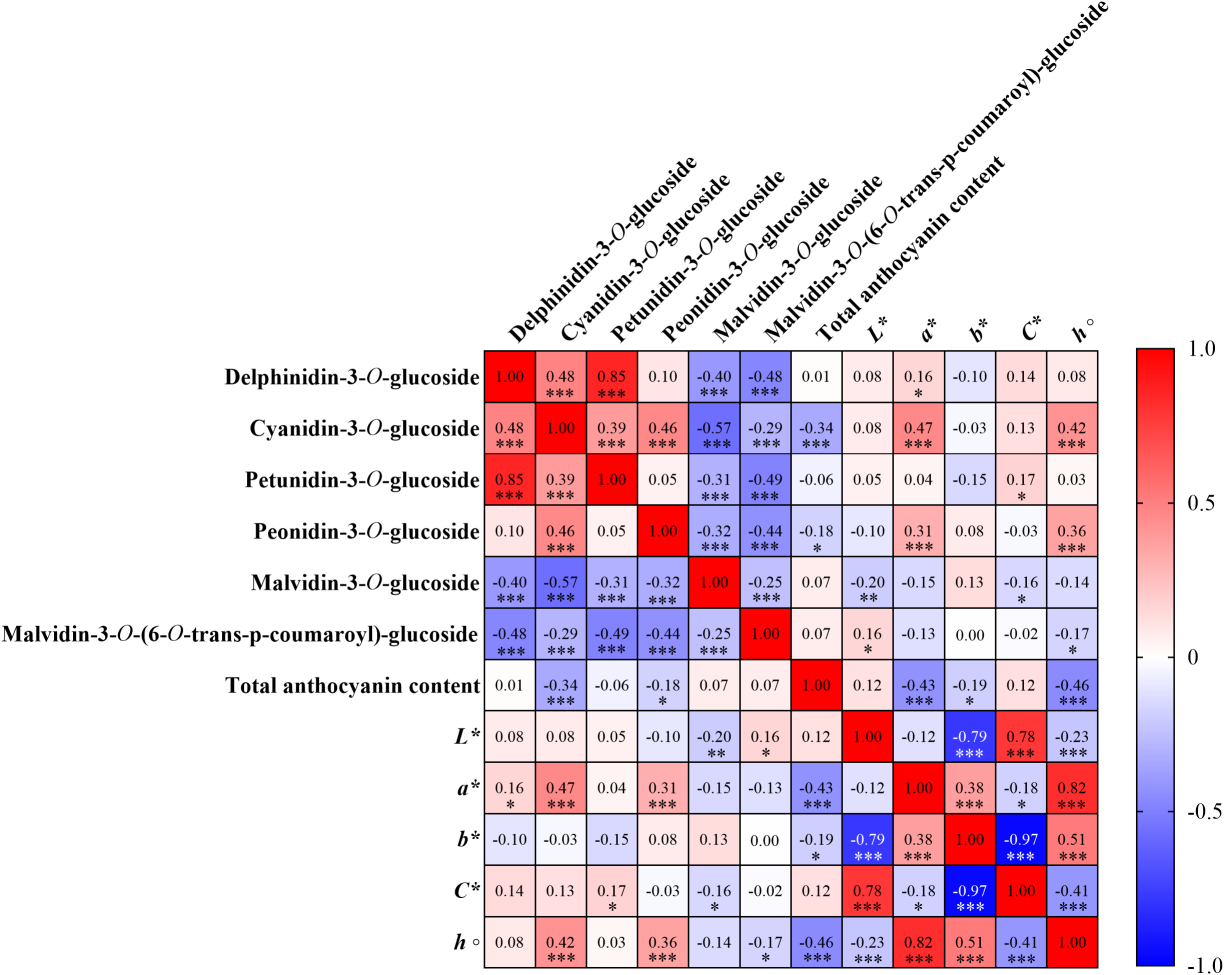
Pearson’s correlation matrix of anthocyanin content and chromatic parameters for a confidence level of 5%. Color scale ranges from red (1) to blue (-1). For statistically significant correlations: *p < .0.05; **p < 0.01; ***p < 0.001.

The Pearson correlation matrix (**Figure 4**) reveals the relationships between the total anthocyanin content, individual anthocyanins, and the CIELAB parameters in all of the red grape varieties under study. Strong positive Pearson correlations coefficients between specific anthocyanins were observed, especially between delphinidin-3*-O-*glucoside and petunidin-3*-O-*glucoside (r = 0.85, *p* < 0.001), while cyanidin-3*-O-*glucoside presented moderate positive correlations with delphinidin-3*-O-*glucoside (r = 0.48, *p* < 0.001) and peonidin-3*-O-*glucoside (r = 0.46, *p* < 0.001). Malvidin derivatives, namely malvidin-3*-O-*glucoside and malvidin-3*-O-*(6*-O-*trans-p-coumaroyl)-glucoside revealed weak correlations with the remaining anthocyanins and in-between both, with values ranging from -0.25 to -0.57 (*p* < 0.001). Nonetheless, malvidin anthocyanins were the only ones exhibiting a non-significant weak positive correlation with the total anthocyanin content (r = 0.07, *p* > 0.05). The color parameters, L*, a*, b*, C*, and *h°*, were, for the most part, found to be weakly correlated with individual anthocyanin content. Nonetheless, a* presented a positive correlation with cyanidin-3*-O-*glucoside (r = 0.47, *p* < 0.001), peonidin-3*-O-*glucoside (r = 0.31, *p* < 0.001) and *h°* (r = 0.82, *p* < 0.001), indicating a contribution of this anthocyanin to the redness of the grape skins, while also being negatively with total anthocyanin content (r = -0.43, *p* < 0.001). Correlations with total anthocyanin content were generally weak, apart from the afore mentioned negative correlation with a*, and the correlation with *h°* (r = -0.46, *p* < 0.001). L* presented negative correlations with b* (r = -0.79, *p* < 0.001), indicating that darker grapes tend to of blueish tint, while also having positive correlations with C* (r = 0.78, *p* < 0.001) and negative with *h°* (r = 0.23, *p* < 0.001). Regarding b*, this parameter presented strong negative correlations with L* (r = -0.79, *p* < 0.001), C* (r = -0.97, *p* < 0.001) and a strong positive correlation with *h°* (r = 0.51, *p* < 0.001).

**Figure 5:**
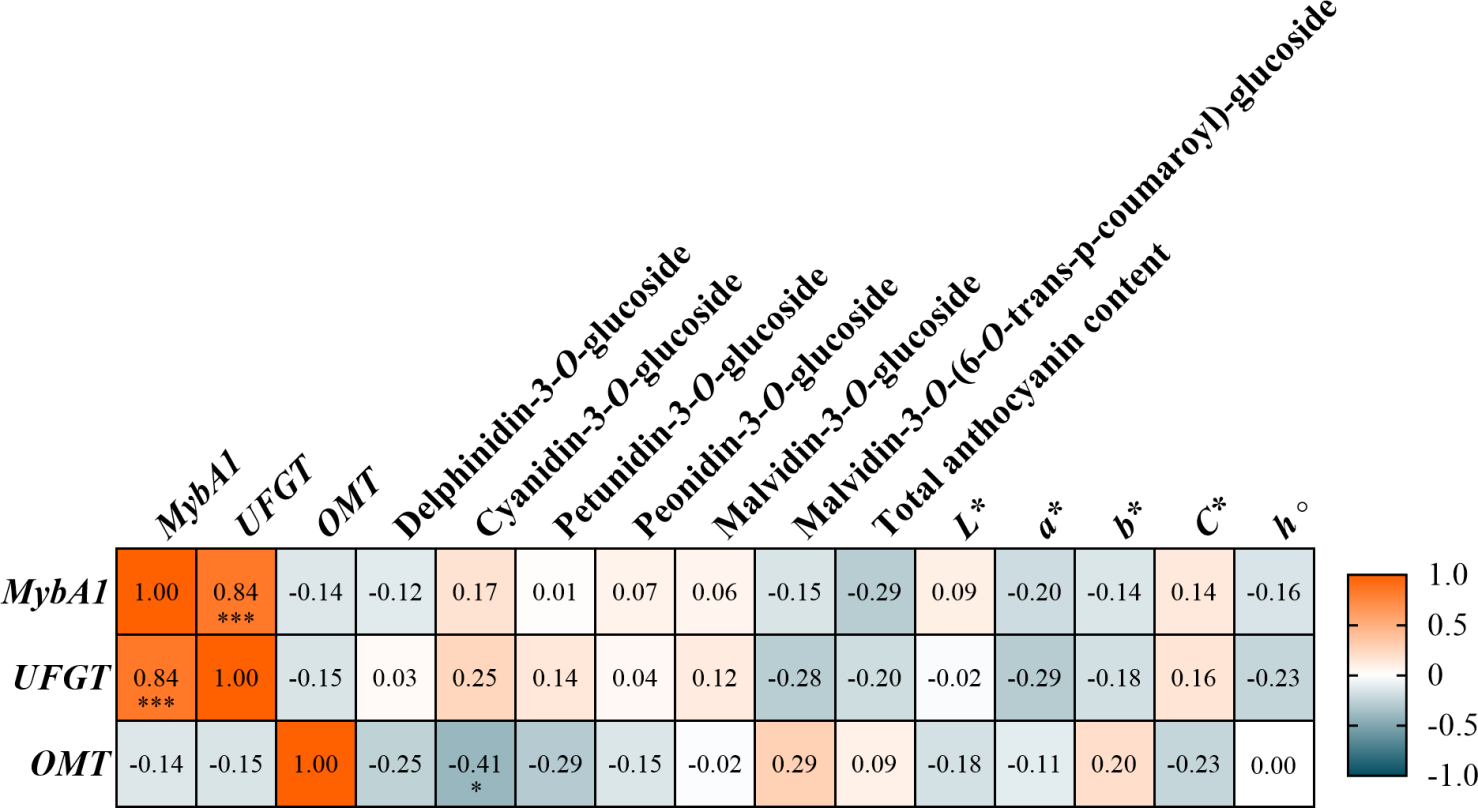
Pearson’s correlation matrix of the genes under study with the anthocyanin content and chromatic parameters for a confidence level of 5%. Color scale ranges from orange (1) to teal (-1). For statistically significant correlations: *p < .0.05; **p < 0.01; ***p < 0.001.

For the six varieties selected to study gene expression, ‘Cabernet Sauvignon’, ‘Marufo’, ‘Touriga Franca’, ‘Touriga Nacional’, and ‘Vinhão’, a Pearson correlation analysis was performed in order to compare gene expression with the remaining parameters (**Figure 5**). A strong positive correlation was observed between *MybA1* and *UFGT* (r = 0.84, *p* < 0.001), indicating a close relationship in their regulatory roles in anthocyanin biosynthesis, while both exhibited non-significant weak negative correlations with *OMT* (r = -0.14 and r = -0.15, respectively). Regarding anthocyanin composition, cyanidin-3*-O-*glucoside was the only one who exhibited a statistically significant weak positive correlation with *UFGT* (r = 0.25, *p* < 0.001). Petunidin-3*-O-*glucoside, peonidin-3*-O-*glucoside, malvidin-3*-O-*glucoside, malvidin-3*-O-*(6*-O-*trans-p-coumaroyl)-glucoside and total anthocyanin content displayed no statistically significant correlations with the genes under study. Furthermore, the same was observed for the CIELAB parameters.

### 3.6 Principal component analysis

**Figure 6:**
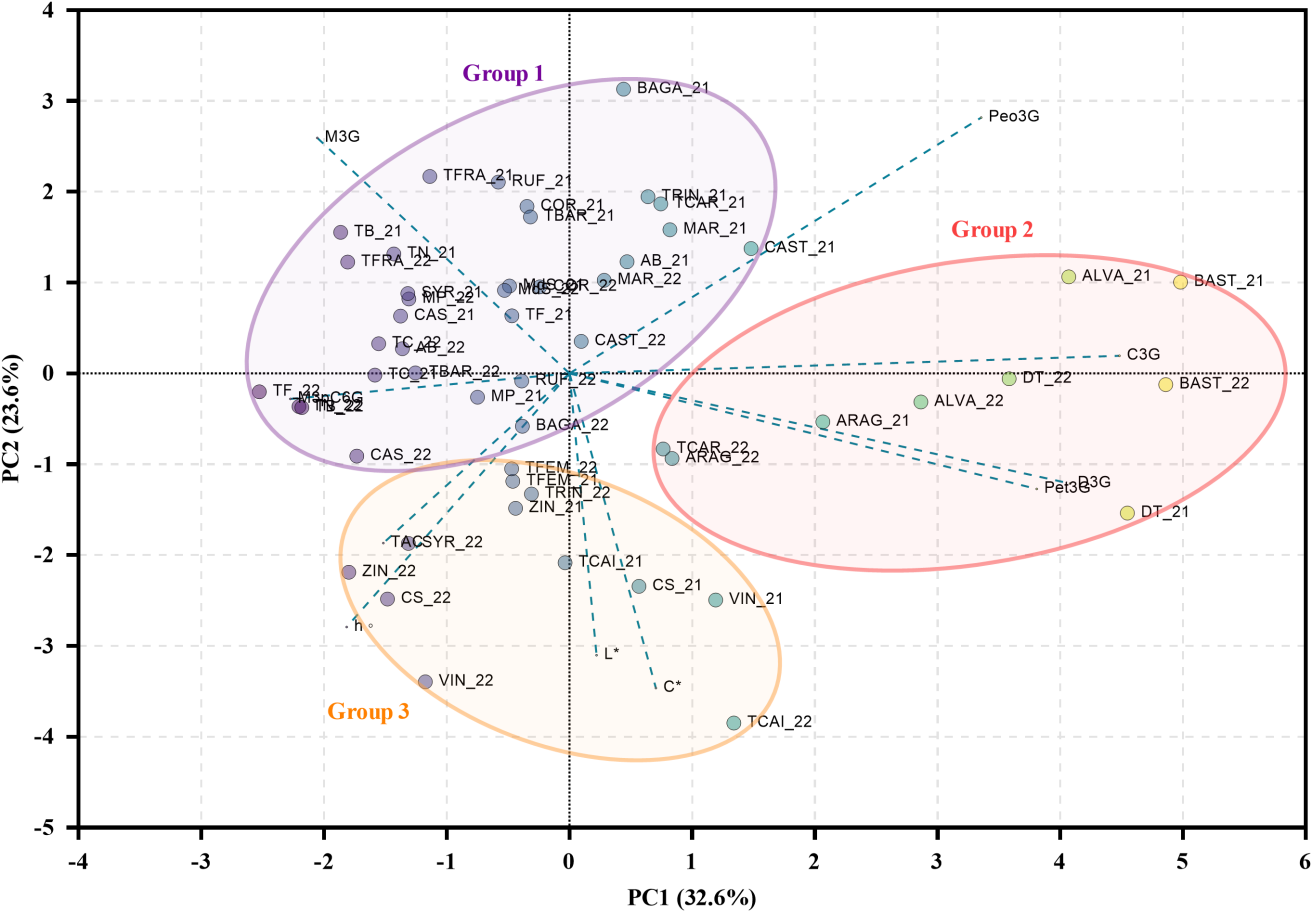
Biplot (PC1 vs. PC2) of the PCA model built with the different anthocyanin-related berry traits. Parameters: D3G, Delphinidin-3-O-glucoside; C3G, Cyanidin-3-O-glucoside; Pet3G, Petunidin-3-O-glucoside; Peo3G, Peonidin-3-O-glucoside; M3G, Malvidin-3-O-glucoside; M3pC6G, Malvidin-3-O-(6-O-trans-p-coumaroyl)-glucoside; TAC, Total Anthocyanin Content. Varieties: AB, Alicante Bouschet; ARAG, Aragonez; ALVA, Alvarelhão; BAGA, Baga; BAST, Bastardo; CAS, Casculho; CAST, Castelão; COR, Cornifesto; CS, Cabernet Sauvignon; DT, Donzelinho Tinto; MAR, Marufo; MdS, Mourisco de Semente; MP, Malvasia Preta; RUF, Rufete; SYR, Syrah; TB, Tinta Barroca; TBAR, Tinta da Barca; TC, Tinto Cão; TCAI, Tinta Caiada; TCAR, Tinta Carvalha; TF, Touriga Franca; TFEM, Touriga Fêmea; TFRA, Tinta Francisca; TN, Touriga Nacional; TRIN, Trincadeira; VIN, Vinhão; ZIN, Zinfandel.

To further explore the structure of the dataset, a PCA was performed using total anthocyanin content, profile, and the CIELAB parameters C*, L*, and *h°* (**Figure 6**). Two principal components (PC) with eigenvalues greater than one were retained, explaining a cumulative variance of 56.2%, with PC1 and PC2 accounting for 32.6% and 23.6%, respectively. PC1 was mainly driven by delphinidin-3-*O*-glucoside, cyanidin-3-*O*-glucoside, petunidin-3-*O*-glucoside, and malvidin-3-*O*-(6-*O*-trans-p-coumaroyl)-glucoside while PC2 was more strongly associated with chromatic parameters, particularly C*, L*, and *h°*, as well as malvidin-3-*O*-glucoside.

Although no strict clustering was observed based solely on year or variety, three main groups of samples were identified. Group 1, located in the upper left quadrant, included varieties such as ‘Touriga Franca’, ‘Rufete’, ‘Tinta Barroca’, ‘Trincadeira’, and was associated with higher levels of malvidin-3-*O*-glucoside and a moderate range of the chromatic parameters. Group 2, positioned on the right side of the plot, included varieties such as ‘Bastardo’, ‘Donzelinho Tinto’, and ‘Alvarelhão’, having stronger associations with cyanidin, peonidin, and delphinidin derivatives, reflecting a prevalence of 3′- and 3′,5′-substituted anthocyanins. Group 3, located in the lower left quadrant, included ‘Vinhão’, ‘Cabernet Sauvignon’, ‘Zinfandel’, and ‘Tinta Caiada’, was generally associated with reduced values of C* and *h°*, indicating distinctive pigmentation traits.

## 4 Discussion

This study provides a comprehensive characterization of 27 red grape varieties cultivated under the same terroir conditions over two consecutive years (2021 and 2022). Among these, we studied 4 international varieties, as well as 13 with limited representation in the Portuguese vineyard area (less than 1%). By examining biochemical and genetic factors, we attempted to elucidate how intrinsic varietal traits and environmental fluctuations influence berry quality, adding to the knowledge needed for improving climate resilience in viticulture.

Total anthocyanin content (TAC) exhibited significant variability across varieties and years, with some varieties presenting high anthocyanin content such as ‘Vinhão’ and ‘Touriga Franca’, while others presented very low amounts, namely ‘Bastardo’ and ‘Marufo’. Despite a general increase in TAC between 2021 and 2022, different behaviors were observed between varieties (**Figure 2**), reflecting both genetic differences as well as environmental influence. In fact, 2022 was considered a warmer and dryer year than 2021, as observed in the climatic data collected in the experimental site (**Figure 1**). An increase in TAC was observed in most varieties in 2022, being significant for in, ‘Casculho’, ‘Mourisco de Semente’, ‘Tinta Barroca’, ‘Tinta da Barca’, ‘Tinto Cão’, ‘Touriga Nacional’, ‘Trincadeira’, and ‘Zinfandel’. This behavior aligns with previous studies, where abiotic stress enhances anthocyanin synthesis, probably as a protection mechanism (Cohen et al., 2008; Tarara et al., 2008; Santesteban et al., 2011; Hochberg et al., 2015; de Rosas et al., 2022; Hewitt et al., 2023; Dou et al., 2024). Considering the shift in temperature and precipitation from 2021 to 2022, this could suggest a possible adaptation to the region’s environmental conditions, as these compounds are known to aid on the mitigation of oxidative stress (Kaur et al., 2023). Furthermore, this resilience may stem from upregulated antioxidant pathways or improved water-use efficiency (Bucchetti et al., 2011; Santesteban et al., 2011).

Despite the general increase, some varieties did decrease in TAC, namely ‘Vinhão’ a high-quality Iberian grape variety known for being rich in anthocyanin content (Dopico-García et al., 2008; Ferreira et al., 2019a; Díaz-Fernández et al., 2022). This behavior could represent a higher susceptibility of a variety to harsher abiotic conditions, either due to a reduction in anthocyanin biosynthesis, or increased degradation (Mori et al., 2007). On the other hand, this phenomenon could be attributed to a higher activity of biosynthetic pathways promoting other stress-related compounds that increase plant stress endurance (Mirás-Avalos and Intrigliolo, 2017). Nonetheless, the fact that ‘Vinhão’ presented a significant decrease in anthocyanin content is an indicator of how the behavior of grapevine varieties grown under the same terroir can be totally different from one another. In fact, Hochberg et al. (2015) observed that the metabolic response to abiotic stress in grapes can be varietal-dependent, leading to different responses on the polyphenolic metabolism. In fact, Zinfandel’ and ‘Tinta Barroca’ showed significant TAC increases in 2022, likely due to stress-induced upregulation of flavonoid pathways (Jeong et al., 2006; Santesteban et al., 2011; Azuma et al., 2012; Torres et al., 2017). These findings emphasize that climate resilience is not uniform across genotypes.

Among all varieties, the lowest TAC was consistently observed in both ‘Bastardo’ and ‘Marufo’ in both years, indicating stability in this metabolic response. However, this makes these varieties less ideal for the production of deeply colored red wines (Sáenz-Navajas et al., 2011; He et al., 2012), without regarding other potential properties.

The strong varietal effect observed in the anthocyanin profiles (*p* < 0.001, **Table 1**) underscores the genetic predisposition of grape cultivars to synthesize distinct anthocyanin profiles. Moreover, total anthocyanin content showed only weak correlations with most individual anthocyanin forms (**Figure 4**), an unexpected outcome that may reflect marked variability in anthocyanin composition among varieties and the contribution of derivatives not individually quantified in the present work. Malvidin-derived compounds were the predominant anthocyanins found in the varieties under study (**Table 1**). This finding aligns with previous studies, as these derivatives are usually found in higher quantity in most varieties of *V. vinifera* (Guidoni et al., 2008; de Rosas et al., 2017; Díaz-Fernández et al., 2022), and are prevalent in wines, where they provide stable color (Lambert et al., 2011). In fact, malvidin-3*-O-*glucoside and malvidin-3*-O-*(6*-O-*trans-p-coumaroyl)-glucoside both presented negative correlation with the remaining anthocyanins, indicating that as malvidin content increases, the remaining compounds tend to decrease. Nonetheless, the high percentage of the acetylated malvidin derivate malvidin-3*-O-*(6*-O-*trans-p- coumaroyl)-glucoside in 2022, for example in ‘Tinta Barroca’ and ‘Touriga Franca’, suggests that warmer conditions may favor the acylation process in order to enhance anthocyanin stability and reduce their degradation due to harsher conditions (He et al., 2010a; Falginella et al., 2012; Gaiotti et al., 2018). Acetylated anthocyanins are considered less extractable during winemaking but can contribute to long-term color stability through copigmentation (Ortega-Regules et al., 2006; Díaz-Fernández et al., 2022). Contrastingly to what was observed in most varieties, Donzelinho Tinto’ and ‘Bastardo’ emerged as outliers with higher levels of cyanidin-3*-O-*glucoside than most varieties, highlighting how anthocyanin profiles can differ. This anthocyanin, along with peonidin and malvidin-3*-O-*glucoside, is usually considered more stable when it comes to wine color (Cabrita et al., 2000; Zhao et al., 2022). Moreover, as observed by Díaz-Fernández et al. (2022), this contrast could also be due to varieties with low content of anthocyanins having a higher amount of cyanidin, while varieties with higher content usually possess more anthocyanins of the end of the biosynthetic pathway. In fact, these authors also observed that ‘Sousón’ (synonym of ‘Vinhão’ in this work) contained a high content of anthocyanins, with a higher percentage of malvidin derivatives. Nonetheless, varieties with low TAC and high cyanidin percentages could still be used in the production of premium wines, especially if their grape quality remains stable in-between seasons, similarly to what was observed in ‘Bastardo’. Delphinidin- and petunidin-3*-O-*glucosides decreased significantly in 2022 for most varieties, which could likely be attributed to a lower activity of flavonoid 3’,5’-hydroxylase, as it is inhibited due to higher temperatures (Azuma et al., 2012) and modulated by water deficit (Castellarin et al., 2007). In fact, delphinidin-3*-O-*glucoside and petunidin-3*-O-*glucoside presented a correlation of r = 0.86 (**Figure 4**), indicating these might accumulate together. Understanding how anthocyanin profile varies between years, especially in the amount of 3′-substituted, 3′,5′-substituted anthocyanins and their acylated and non-acylated forms could be a powerful tool for future proofing viticulture (Díaz-Fernández et al., 2022). In this study, Touriga Nacional’ exhibited a stable behavior across years, suggesting that the abiotic stress endured was probably not enough to promote drastic changes, making it a potential variety to cultivate in hotter and drier climates. Despite this, the profile variability observed in the analyzed grape varieties emphasizes the effects of both genetic and environmental factors influencing anthocyanin profiles.

Berry quality directly affects the wines produced, especially in terms of color perception. It is therefore important to understand how differences and changes in anthocyanin profile across varieties can dictate these parameters (Liang et al., 2011). In general, most varieties presented higher values of L* in 2022, alongside shifts in a* towards more reddish hues and b* towards more blue tones (**Table 2**). The Pearson correlation performed revealed that anthocyanin content and profile directly influenced grape color. In fact, the weak correlation between L* and total anthocyanins confirms that higher pigmentation not always leads to darker berries, a critical factor in wine consumer perception (Rolle and Guidoni, 2007). Moreover, the general shift towards blueish tones (decrease in b*) in 2022 might have been driven by the previously discussed increase of malvidin-acylated forms in most varieties (**Table 1**) (Liang et al., 2011). Large shifts towards red (increases in a*) were also observed in some varieties, namely ‘Bastardo’, which could be attributed to the changes in anthocyanin profile, as this variety presented a large percentage of cyanidin-3*-O-*glucoside, and both parameters are highly correlated (r = 0.47).

C* and *h°*further highlighted the role of anthocyanins in influencing the color variations observed in grape berries. The significant C* increases in 2022 align with the increase in stable acylated anthocyanins, indicating these might intensify color saturation (Boulton, 2001; Gomez et al., 2009). Meanwhile, *h°* was a lot more heterogeneous, indicating a higher variability among varieties and years, despite being most likely determined by the total anthocyanin content. Further study on these relationships could improve the understanding of color profile and anthocyanin composition in different grape varieties, aiding on the prediction of the visual and sensory qualities of the resulting wines.

Gene expression analysis of three key anthocyanin biosynthesis genes, *MybA1*, *UFGT*, and *OMT* provided insights into the anthocyanin variability of five varieties, namely: ‘Cabernet Sauvignon’, ‘Touriga Franca’, and ‘Touriga Nacional’, which were selected based on their behavior and prevalence in the viticultural sector; and ‘Marufo’ and ‘Vinhão’, which presented contrasting results for TAC (**Figure 2**). A strong correlation between *MybA1* and *UFGT* gene expression (r = 0.84, **Figure 5**) reflects their synergistic role in anthocyanin biosynthesis, as *MybA1* regulates the transcription of several genes of the anthocyanin biosynthetic pathway, including *UFGT*, which catalyzes the glycosylation of unstable anthocyanidins into stable pigments (Poudel et al., 2021; Yan et al., 2021). *MybA1* expression results revealed no statistically significant differences between years for any variety, although slight decreases were observed for ‘Cabernet Sauvignon’ and ‘Touriga Franca’ in 2022. Nonetheless, when comparing both years, *UFGT* was observed to be significantly downregulated in ‘Cabernet Sauvignon’ and ‘Touriga Franca’ in 2022 (**Figure 3**), despite not being reflected on the TAC of both varieties (**Figure 2**). These results could indicate that either the lower abiotic stress in 2021 allowed for a prolonged synthesis of anthocyanins, or the higher temperatures and dryer conditions of 2022 led to an earlier suppression of *UFGT*. In fact, *MybA1* and *UFGT* activity are usually at their peak during veraison (Ali et al., 2011; Pastore et al., 2017; Ferreira et al., 2019a), while this study evaluated their activity at harvest. Nonetheless, the fact that both ‘Cabernet Sauvignon’ and ‘Touriga Franca’ had higher expression of these genes in 2021 might indicate that the harsher conditions of 2022 could have suppressed *UFGT* activity (Pastore et al., 2017).

The *OMT* gene leads to the synthesis of *O*-Methyltransferase, which is responsible for the methylation of delphinidin into petunidin, delphinidin into malvidin, and petunidin into malvidin. Moreover, a similar reaction occurs turning cyanidin into peonidin (Ma et al., 2021). The correlations analysis between *OMT* gene expression and individual anthocyanins did not account for any noticeable differences (**Figure 5**). In fact, most varieties presented low levels of *OMT* in both years, with the only exception being ‘Touriga Nacional’ in 2021 (**Figure 3**). Nonetheless, this variety presented no significant changes in anthocyanin profile across both years. *OMT* can lead to the accumulation of malvidin derivatives, as well as peonidin derivatives (Hugueney et al., 2009; He et al., 2010b) and should therefore be very active in *V. vinifera* grape berries. However, its function could very well be limited to the fruit development stage, in similarity to other anthocyanin biosynthesis genes (Vallarino et al., 2014).

Finally, PCA results (**Figure 6**) suggested a partial clustering of the varieties into three main groups based on their anthocyanin composition and chromatic traits. Moreover, the proximity of each variety across both years reinforced the existence of genotype-dependent environmental interactions, supporting the relevance of varietal selection for climate adaptation.

This comprehensive study of anthocyanin content and profiles in 27 red grape varieties cultivated in Portugal represents a significant contribution to our understanding of grapevine diversity and its potential role in adapting to climate change. By providing detailed characterization of these varieties, this research offers valuable insights for viticulturists, winemakers, and researchers working on climate change adaptation in viticulture. As climate change continues to pose challenges for the agricultural sector, and particularly to viticulture, researching the variability among grapevine varieties becomes increasingly important in ensuring the sustainability and quality of wine production. Moreover, this study highlights the value of preserving and studying local grape varieties, which may possess unique characteristics that could prove advantageous under changing environmental conditions. This is particularly true when we look at diversity among Portuguese varieties such as ‘Touriga Nacional’, ‘Touriga Franca’ and ‘Trincadeira’ which revealed increases in anthocyanin content, and varieties such as ‘Bastardo’ and ‘Marufo’, which presented a consistent lower amount of anthocyanin content, but could be preferable for being distinctive.

Overall, this study contributes valuable knowledge regarding the anthocyanin composition of grape varieties and emphasizes the importance of understanding both genetic backgrounds and environmental influences for optimizing grape cultivation and wine production. Moreover, it underscores the importance of studying varietal behavior in order to improve varietal selection in viticulture, as lesser-known varieties can have potential in contributing to the resilience and adaptability of the wine industry in the face of climate change. Future research should focus on extended observations spanning more years, in order to further deepen this knowledge and truly access intervarietal and annual variability, as well as elucidate the molecular mechanisms underlying anthocyanin biosynthesis and exploring how different viticultural practices can enhance desirable traits associated with wine quality.

## Data Availability

The original contributions presented in the study are included in the article/**Supplementary Material**. Further inquiries can be directed to the corresponding authors.
